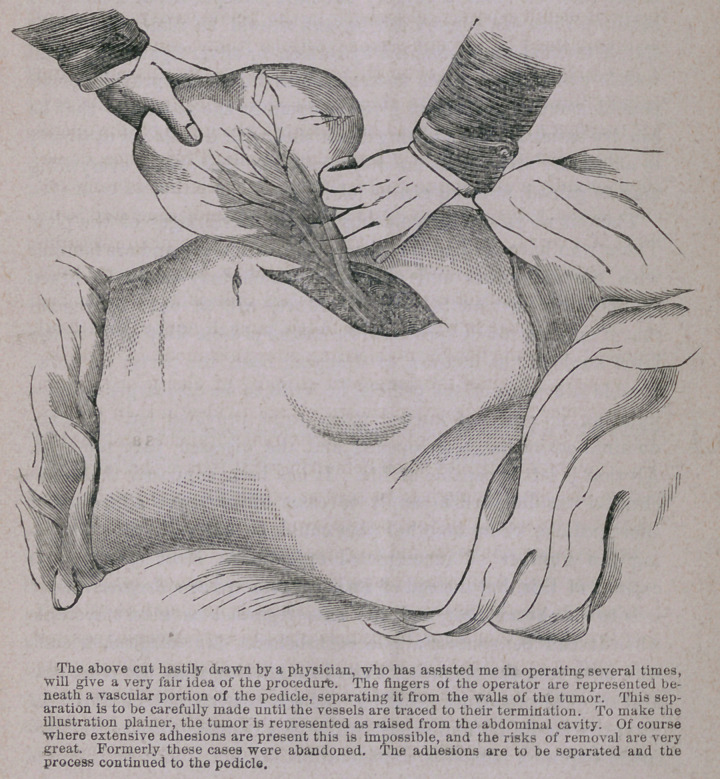# Ovariotomy by Enucleation, What It Is and How to Do It

**Published:** 1875-06

**Authors:** Julius F. Miner

**Affiliations:** Professor of Special Surgery in Buffalo Medical College


BUFFALO

### Medical and Surgical Journal.

VOL XIV. JUNE, 1875. No. 11.

### Original Communications.

---:o:---

ART. I.—*Ovariotomy by Enucleation. What it is, and how to do it.* By JULIUS F. MINER, M. D., Professor of Special Surgery in Buffalo Medical College.

It is now about six years since I first announced to the profession that the Ovarian Tumor could be removed by Enucleation, and invited my professional friends to make trial of the proposed plan, describing as well as I could what I had done, and the conclusions I had formed. The idea that a tumor having such large arterial supply could be removed without clamp, ligature or cautery, though at first startling, was very readily accepted, and both in this country and in Europe, successful trial has led many of the most distinguished operators to not only make trial of it, but to speak of it in high terms of commendation, until now it is one of the established and acknowledged methods of operation. By the numerous reports and papers upon the subject, I discover that the exact manner of enucleation is not yet distinctly understood, some have spoken of *clamp* after enucleation, others have spoken of *cutting*, the very thing it is designed to avoid. Others still have limited the detachment of the pedicle to two or three inches above its base, thus showing me that I have never been fully understood in the method of removing ovarian tumors by enucleation, a plan which my experience convinces me, if properly understood and executed, possesses advantages over all others and is of almost universal application. It is well known that the ovarian tumor is surrounded by a peritoneal covering, that the pedicle, proper, usually divides into three or four parts passing up over the walls of the tumor in bands of variable width containing vessels often of large size, which with connective tissue, make a band which passes over the walls of the cyst, gradually diminishing in thickness and in the size of the vessels it contains, until finally it is lost in a simple thickened part of peritoneal covering. The peritoneal covering is not closely attached to the cyst, but separates readily the same that the peritoneum separates elsewhere in the pelvic cavity, being immediately lined by the sub-serrous cellular tissue, thus no vessels of any considerable size enter the cyst. This cyst separates from its attachments with remarkable readiness, so much so that in several instances it is reported to have escaped the grasp of the operator and fallen spontaneously from the pedicle; Providence, or accident plainly indicating the natural and proper method of removal. The capillary vessels thus broken do not bleed, the band contracts and corrugates the larger trunks, while the broken off capillary vessels thus separated ooze only a little for a minute or two, a dry napkin applied for a short time is all that is required. The fear of hemorrhage is wholly unfounded, and I now say without hesitation that the danger of bleeding after this mode of proceedure is vastly less than the danger of slipping of clamp or ligature in the former methods, when the vessels are divided in their trunks. Here they are separated only in their extreme branches and cannot bleed, do not give troublesome hemorrhage; it is rare that any vessels are torn large enough to be seen as vessels or points of hemorrhage, and torsion is all that can be required in almost any case If care is taken not to wound the vessels with either trocar, knife or scissors, there will be no hemorrhage. The tumor being thus removed the operation is completed. There is no clamp to be used, there is nothing to clamp, the pedicle is not to be treated, it requires no attention except careful manipulation and resting back in its original place as near as possible, if the usual conditions are present no drainage is necessary, the incision may be closed as perfectly as possible. These bands are to be grasped where they commence to diverge with the hand and raised from the cyst, tracing out the band to its termination often nearly around to the opposite side. The idea is not that the cyst is to be separated from a capsular investment, as some tumors are enucleated. It is only to be separated from its vascular supply which is contained in these bands. Any other attachments are to be separated in the usual manner. Care is necessary not to wound or divide the vessels in their trunks, and although the attachment will sometimes be found very strong at points, it can be forced off, or even with care a small piece of the cyst may be left attached to the pedicle, and no harm can result from it, it has vascular supply, and is living tissue, like all the rest of the pedicle which is left. Nothing remains to suppurate, become encysted or to be absorbed or otherwise provided for.

Again this plan may be tried first and no harm result from it. If for any reason it should be deemed impracticable, a clamp, the most unsurgical appliance in the world, can be equally well applied. The pedicle can subsequently be burned or tied with ligature equally well as if enucleation had not been tried, for I am going to say that few rules in surgery but have exceptions, and though I believe all ovarian tumors can be, and should be removed by this simple method, supplemented by torsion or silver ligature to small vessels which bleed, when necessary, still I desire to provide for all possible contingencies, and give the operator the assurance that he can try enucleation, and being dissatisfied with it he is yet at liberty to adopt any other plan he may prefer, so that while everything may be gained, nothing can be lost.

There is no reason for pointing out the advantages of the plan. Those who have studied the history of ovariotomy and are familiar with the difficulties and objections which may fairly be urged against all former methods of procedure, will at once apprehend that if enucleation is successful he has no pedicle keeping open the lower angle of the incision, or dragging open the parts; no unfavorable adhesions of the pedicle, no wires to be discharged by suppuration, no crusts of burned tissue to be provided for. The abdominal cavity has been opened and the diseased part removed. All that is left is capable of life. It has been supposed that enucleation was designed to apply to cases of extensive adhesions or short pedicle, where no other plan could be adopted, thus lessening the number of incompleted operations. Most clearly it is capable of this, but instead of its being reserved as a *dernier ressort* it is to be chosen first, and the case regarded as most favorable when it can be successfully accomplished. My surgical friends who have seen the operation unite in regarding it as the most natural surgical procedure possible. To see it is to be convinced of its entire feasibility and safety, while its advantages are too apparent to require a moments consideration.

Since writing the above, two cases, illustrating every point connected with *Enucleation*, have fallen under notice.

Prof. James P. White operated by enucleation in Oneida county, and desires me to say, he has now adopted the method in four cases, and thinks "this method to be *chosen first*, and if vessels bleed, or any conditions are found, making it necessity to employ former methods, to do so after trial of enucleation." Wednesday, June 23, I had also opportunity to satisfactorily demonstrate every position taken in this paper, to nearly the entire profession of Buffalo, by an operation upon a private patient, Mrs. Cobb, from Penn., removing by the above method an ovarian cyst of great size, without any vessel requiring even torsion, and without any hæmorrhage at all. It was one of those cases formerly abandoned by surgeons as immovable, on account of adhesions, being closely adherent to the walls of the abdomen on all sides. After cutting down upon the cyst proper, it was easily pulled out of its bed; no pedicle being found which would make it impossible to remove it by any of the former methods. Mrs. Cobb died the third day in convulsions.

But enucleation is not for these desperate cases *alone*; it is applicable to all cases, and as Dr. White fairly states, is to be chosen first, other plans adopted, after trial of this.

## Figures and Tables

**Figure f1:**